# Functional Defect of Neutrophils Causing Dermatophytosis: Case Report

**DOI:** 10.3390/jof6040238

**Published:** 2020-10-22

**Authors:** Rosemeire N. Constantino-Silva, Sandro F. Perazzio, Nicolas de Albuquerque Weidebach, Anete S. Grumach

**Affiliations:** 1 Clinical Immunology, Faculdade de Medicina, Centro Universitario Saude ABC, Andre 09060-870, Brazil; meire_biol@yahoo.com.br (R.N.C.-S.); nicolas.weidebach@hotmail.com (N.d.A.W.); 2 Division of Rheumatology, Escola Paulista de Medicina, Universidade Federal de Sao Paulo, Sao Paulo 04023-062, Brazil; sperazzio@yahoo.com.br; 3 Fleury Laboratories, Sao Paulo 04023-062, Brazil

**Keywords:** neutrophil, myeloperoxidase, *Trichophyton*, fungal infection, NCF1, chronic granulomatous disease

## Abstract

**Background**: NADPH-oxidase and myeloperoxidase (MPO) play an important role on defense against pathogenic microorganisms. Defects on these mechanisms have been described in association with recurrent infections due to such as *Staphylococcus aureus* and *Candida albicans.* We describe a patient with partial disturbance of intracellular microorganism destruction clinically manifested by recurrent fungal infection. **Case report and results:** A 58-year-old male rural farmer has suffered with superficial mycosis affecting hands, nails and right ankle persisting for 20 years. He was treated with several antifungal drugs with no improvement. Mycological scraping isolated *Trichophyton rubrum*. Immunological evaluation showed impaired T cell proliferation to *Candidin* and impaired neutrophil burst oxidative after specific stimulation with *Candida albicans*. The patient’s DNA was extracted from peripheral blood leukocytes for whole exome sequencing (WES) analysis. Two heterozygous variants of undetermined significance were screened accordingly: (1) *MPO* A332V (c.995G>A; rs28730837); and (2) *NCF1* G83R (c.247G>A; rs139225348). **Conclusions:** Functional leukocyte evaluation with heterozygous variants in MPO and NCF1 suggest that these defects were associated with the susceptibility to dermatophytosis in our patient. We have developed a fast, effective and safe trial for screening individuals with yeast infections.

## 1. Introduction

Reactive oxygen species (ROS) production is one of the main cytotoxic mechanisms of phagocytes described so far. [1] Nicotinamide adenine dinucleotide phosphate (NADPH)-oxidase is a multicomponent enzymatic complex constituted by two membrane-bound components, gp91 phox and p22 phox, and four cytosolic subunits, including the small GTPase Rac1/2, p40 phox, p67 phox, and regulatory p47 phox (also known as NCF1) [2,3]. NADPH-oxidase plays a pivotal role on ROS production by converting oxygen into a superoxide radical or hydrogen peroxide, albeit the latter may be also produced by superoxide dismutase activity. Loss-of-function mutant NADPH-oxidase subunits impair electron transport system and, in turn, ROS production, which interfere on leukocyte’s ability to destroy phagocytosed microorganisms, culminating with the emergence of chronic granulomatous disease (CGD) [4,5,6].

Myeloperoxidase (MPO) is the main component of neutrophil azurophil granules, although also found in monocytes and eosinophils [7,8]. As a lysosomal hemoprotein, it catalyzes the production of highly reactive radicals such as hypochlorous acid, from hydrogen peroxide and halogen ions, thus playing an important role on defense against pathogenic microorganisms [7,8]. MPO deficiency is a relatively common human genetic defect (1:2000–4000 cases) [9] and may impair bactericidal and fungicidal capacity of polymorphonuclear leukocytes (PMN), mainly against microorganisms such as *Staphylococcus aureus* and *Candida albicans* [10,11]. This piece of data reaffirms the important role of superoxide dismutase and myeloperoxidase interplay within the oxidative-dependent microbicidal killing in neutrophil lysosomes [12].

Taken altogether, the above data demonstrated the relevance of neutrophil oxidative burst on innate response against pathogens. Herein, we describe a patient with partial disturb of intracellular microorganism destruction clinically manifested by recurrent fungal infection.

## 2. Case Report

**Figure 1:**
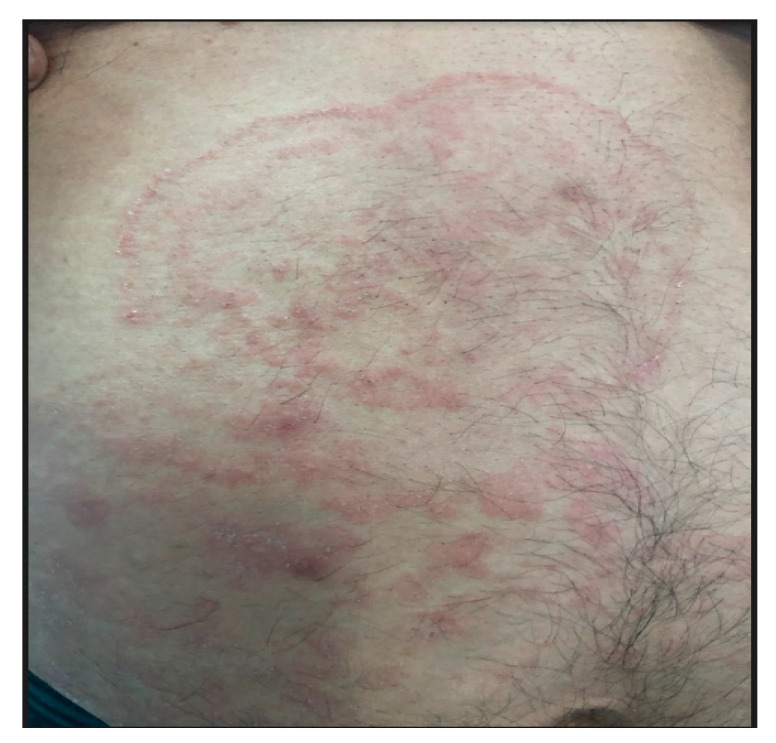
Cutaneous lesions caused by *T. rubrum* affecting the patient.

**Figure 2:**
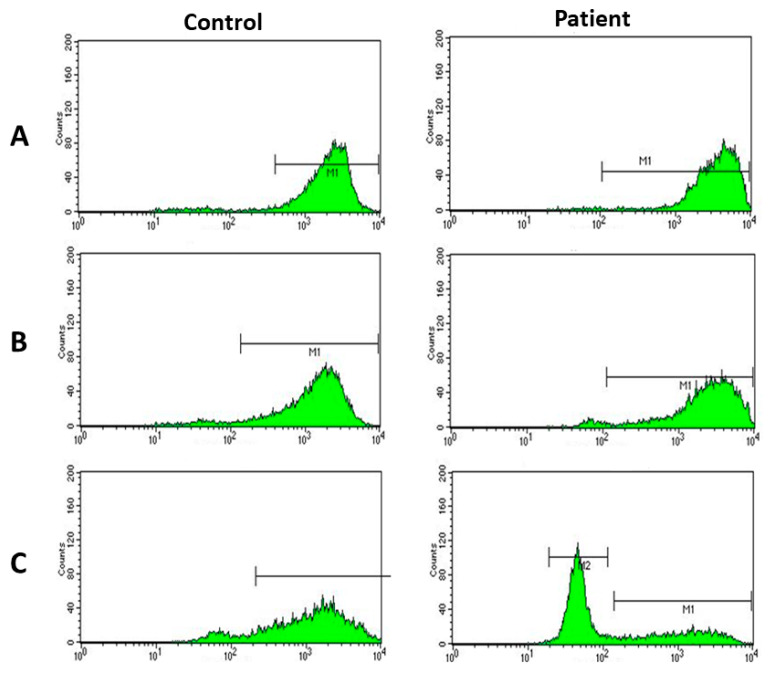
Dihidrorodamine test (DHR) in the control and the patient with Dermatophytosis. (**A**) DHR without stimulus; (**B**) DHR stimulated with *S. aureus*; (**C**) DHR stimulated with *Candida*.

A 58-year-old male rural farmer has suffered with superficial mycosis affecting hands, nails and right ankle persisting for 20 years. A 20 × 15 cm desquamative pruritic liquenified fungal well delimited plaque with regular and thin erythematous borders have recently ascended to inguinal and abdominal areas (Figure 1). Simultaneously, the patient also presented a similar 10 × 10 cm fungal lesion on his upper right paravertebral dorsum. The patient has been receiving long-lasting unsuccessful therapy with griseofulvin (1990–2000; 2016–2018), terbinafine (1985–1990; 2001–2015) and itraconazole (>2018). On the other hand, his brother presented similar symptoms, although was responsive to standard antifungal treatment. No additional relevant family history or consanguinity was observed. Mycological scraping isolated *Trichophyton rubrum*. Due to the absence of visceral fungal history, an upper gastrointestinal endoscopy was also performed and confirmed no invasive fungal infection. Immunological screening (Table 1) showed deficient T cell proliferative response to specific stimuli and impaired neutrophil burst oxidative after specific stimulation with *Candida albicans* (Figure 2A–C). Patient signed consent form and the protocol was approved by the local ethical committee (CAAE 33911520.5.0000.0082, 30/07/2020).

The patient’s DNA was extracted in a clinical setting from peripheral blood leukocytes for whole exome sequencing analysis. Exome capture was conducted in a clinical setting as per the manufacturer’s instructions. Sequencing was performed on an Illumina NextSeq platform and exome datum was aligned to the GRCh37/hg19 reference genome using Burrows–Wheeler Aligner (BWA). Variants were identified using the Genome Analysis ToolKit (GATK) protocol and annotated using ANNOVAR, with a minimum of 95% of target bases covered at >10×. Mitochondrial genome and copy number variants were not studied. Variants were interpreted as per a consensus of two different analytical softwares: an in-house-developed analysis platform (GTAC) and a commercially available diagnostic decision support platform by Emedgene Technologies LTD (Tel-Aviv, Israel) (www.emedgene.com). At least two parallel analyses were performed to preselect variants considering allele frequency <1%, functional impact, clinical relevance of gene harboring the variant, relevant reports from databases (such as ClinVar, HGMD) and the literature. These preselected variants were then discussed in a board with three experts and only selected variants underwent Sanger confirmation. Variants were classified according to ACMG guidelines with assistance of a third-party ACMG calculator by Saphetor SA (Lausanne, Switzerland) (www.varsome.com).

**Figure 3:**
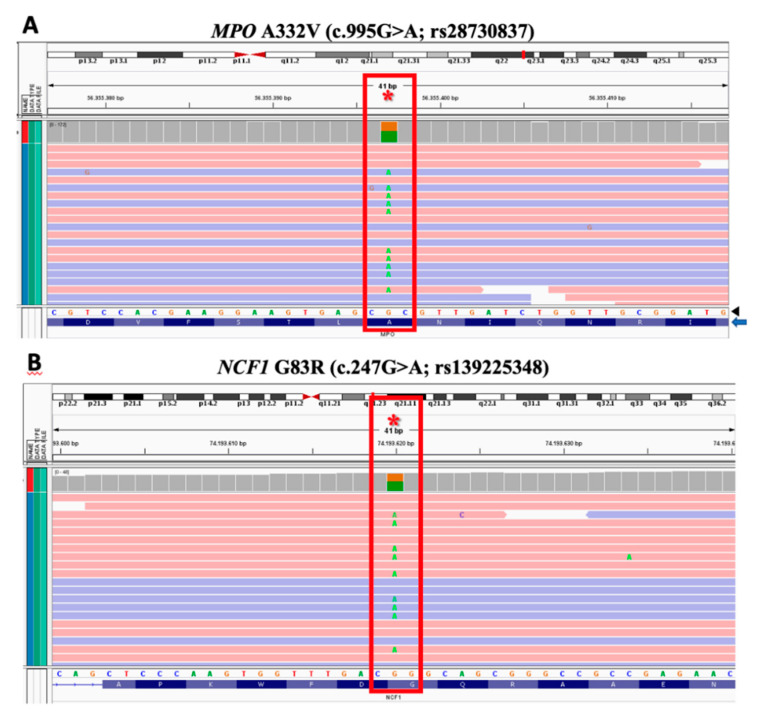
Whole exome sequencing alignment showing oxidative burst-related candidate heterozygous gene variants (red star): (**A**) *MPO* A332V (c.995G>A; rs28730837) and (**B**) *NCF1* G83R (c.247G>A; rs139225348). Green: heterozygous switched base pair (approximately half of acquisition readouts); right bottom panel arrowhead: expected DNA sequence; right bottom panel blue arrow: expected amino acid sequence.

Overall, 58,553 variants were observed. Only 1139 of those have been previously described in less than 5% of healthy controls, among of which 960 were identified in coding regions or exonic boundaries and 296 were synonymous. Finally, 240 were considered for medical analysis and, per the patient’s clinical phenotype, only two heterozygous variants of undetermined significance were screened accordingly: (1) *MPO* A332V (c.995G>A; rs28730837; Figure 3A), whose prevalence in controls was estimated in 4.28% (GnomAD), 1.30% (ExAC) and 0.46% (1000 G); and (2) *NCF1* G83R (c.247G>A; rs139225348; Figure 3B), whose prevalence in controls was estimated in 1.36% (GnomAD), 0.88% (ExAC) and 0.36% (1000 G).

## 3. Discussion

Phagocytes play a particularly important role in fungal infections, mainly against *Candida albicans* and *Aspergillus fumigatus*. Individuals who have primary or secondary neutrophils defects may present refractory invasive fungal infections [13]. MPO deficiency is the most common neutrophil biochemical defect and have been associated with recurrent fungal infections, especially candidiasis, and diabetes mellitus in 5% of individuals [14]. Despite the possible MPO hyperglycosylation secondary to high concentrations of plasmatic glucose, culminating with negative modulation of enzymatic activity, one can never rule out whether this loss-of-function defect may also be resultant of a primary conformational protein defect [15]. An association between MPO deficiency and infection is still unclear; therefore, it has been suggested that MPO deficiency itself does not increase susceptibility to infections, but may synergistically work with other conditions to decrease the immune defenses against microorganisms [16]. On the other side, McCarthy & Dahl evaluated if the myeloperoxidase-hydrogen peroxide (H202) halide system of neutrophils could inhibit fungal growth, and the N-acetylglucosamine assay was used to measure fungal growth in the presence of various components of that system [17]. An intact myeloperoxidase-H202 halide system significantly decreased the radioactive counts associated with growth of *T. rubrum*. The addition of catalase inactivated the H202 and restored fungal growth, whereas adding heat-inactivated catalase dropped counts back to baseline. In conclusion, fungal growth was inhibited even in the absence of cell-mediated immunity [17]. This activity may prevent fungal sepsis. Additional studies have found an association between MPO deficiency and pustular candida dermatitis [18] or systemic infections (candidiasis and bacteremia) in nondiabetic MPO-deficient patients [19,20], which strengthens MPO primary impairment hypothesis.

NADPH-oxidase subunit p47 phox/NCF1 is considered the complex assembler, since it coordinates the interaction of the five other subunits, allowing multiprotein complex activation [21]. While gp91 phox and p22 phox are the central docking site for the cytosolic components, p47 phox/NCF1 is the subunit responsible for transporting the whole cytosolic complex (p67 phox-p40 phox-GTPase Rac1/2) to the docking site during NADPH-oxidase activation [22,23,24]. Although X-linked gp91 phox-deficient CGD responds for approximately 70% of cases, p47 phox/NCF1 deficiency is the main autosomal recessive form (approximately 25%). Moreover, the importance of p47 phox/NCF1 in host defense was also demonstrated in a p47 phox/NCF1 knockout mice [25]. These mice developed lethal infections and granulomatous inflammation like those encountered in human CGD patients [21].

Our study originally demonstrates two heterozygous variants harbored in distinct genes driving a phagocyte oxidative burst defect and possibly inducing a CGD-like phenotype, as per chronic fungal infection susceptibility. To our best knowledge, NCF1 deficiency represents an autosomal recessive condition without previous association of a similar clinical phenotype on heterozygous mutated patients. Despite both *NCF1* G83R and *MPO* A332V variants are still considered of “undetermined significance” as some asymptomatic carriers are reported, some studies have respectively associated them to inflammatory bowel disease [26] and recurrent infections with MPO deficiency [16,27,28]. Interestingly, a male subject with partial MPO deficiency carrying the same single heterozygous *MPO* A332V mutation described here was previously reported [16]. Additionally, *MPO* A332V was associated with lower levels of MPO in monocytes, demonstrating deleterious enzymatic function in patients carrying this variant [27]. Although instigating, we were not able to rule out a possible digenic inheritance pattern in our patient, since our study did not have enough power. Further studies are being designed with cells donated by the same patient reported herein to address this important question.

We also found impairment in T cell proliferation with specific antigens, including Candidin. It is possible that mannan from *T. rubrum* might have an activity similar to mannan from *C. albicans* inhibiting mitogen induced lymphoproliferation [29,30].

Laboratory tests to investigate neutrophil response against *C. albicans* are scarce due to cell viability constraints. We have developed a fast, effective and safe trial for screening individuals with yeast infections. A little amount of sample is necessary, which is a great advantage, since routinely functional tests usually require a large amount of blood.

## 4. Conclusions

Clinical manifestations are very important to guide immunological evaluation. Functional tests were essential to demonstrate the susceptibility to fungal infection and molecular diagnosis has to be considered in the face of heterozygous mutations found.

## Figures and Tables

**Table 1:** Immunological evaluation of the patient with *T. rubrum* infection.

Parameters	Patient	Reference Range
IgG (mg/dL)	1070	384–1200
IgM (mg/dL)	85	40–230
IgA (mg/dL)	339	22–176
IgE (UI/mL)	3829	<100
Leukocytes (cells/mm^3^)	12,960	6000–11,000
Isoagglutinin	Anti-B: 1/32	>1/4
Serology for rubella	IgG + IgM-	Positive IgG
Serology for CMV	IgG + IgM-	Positive IgG
Antipneumococcal antibodies	<0.5 for 7 serotypes	Positive response
B cells cells/mm^3^ (%)	117 (8.04)	161–979 (12.8–38.4)
T CD3+ cells/mm^3^ (%)	1187 (81.24)	605–2460 (60–87)
T CD4+ cells/mm^3^ (%)	787 (53.85)	493–1666 (32–61)
T CD8+ cells/mm^3^ (%)	400 (27.4)	224–1112 (34–43)
Proliferative response to PHA (s.i.) *	24.1	Control = 128.9
Proliferative response to CMV (s.i.) *	5.7	Control = 99.8
Proliferative response to tetanus toxoid (s.i.) *	0.5	Control = 50.1
Proliferative response to candidin (s.i.) *	4.6	Control = 80.5
NK cells cells/mm^3^ (%)	134	73–654 (4–28)
C3 level (mg/dL)	105	88–165
C4 level (mg/dL)	27	14–44

* stimulation index.

## References

[1] Roos D. (2019). Chronic Granulomatous Disease. Methods Mol. Biol..

[2] Abid M.R., Spokes K.C., Shih S.C., Aird W.C. (2007). NADPH oxidase activity selectively modulates vascular endothelial growth factor signaling pathways. J. Biol. Chem..

[3] Lambeth J.D., Kawahara T., Diebold B. (2007). Regulation of Nox and Duox enzymatic activity and expression. Free Radic. Biol. Med..

[4] Curnutte J.T. (1993). Chronic granulomatous disease: The solving of a clinical riddle at the molecular level. Clin. Immunol. Immunopathol..

[5] Winkelstein J.A., Marino M.C., Johnston R.B., Boyle J., Curnutte J., Gallin J.I., Malech H.L., Holland S.M., Ochs H., Quie P. (2000). Chronic granulomatous disease. Report on a national registry of 368 patients. Med. Baltim..

[6] Segal B.H., Leto T.L., Gallin J.I., Malech H.L., Holland S.M. (2000). Genetic, biochemical, and clinical features of chronic granulomatous disease. Med. Baltim..

[7] Hansson M., Olsson I., Nauseef W.M. (2006). Biosynthesis, processing, and sorting of human myeloperoxidase. Arch. Biochem. Biophys..

[8] Lau D., Baldus S. (2006). Myeloperoxidase and its contributory role in inflammatory vascular disease. Pharmacol. Ther..

[9] Nauseef W.M. (1986). Myeloperoxidase biosynthesis by a human promyelocytic leukemia cell line: Insight into myeloperoxidase deficiency. Blood.

[10] Cech P., Papathanassiou A., Boreux G., Roth P., Miescher P.A. (1979). Hereditary myeloperoxidase deficiency. Blood.

[11] Edwards S.W., Hart C.A., Davies J.M., Pattison J., Hughes V., Sills J.A. (1988). Impaired neutrophil killing in a patient with defective degranulation of myeloperoxidase. J. Clin. Lab. Immunol..

[12] Hampton M.B., Kettle A.J., Winterbourn C.C. (1996). Involvement of superoxide and myeloperoxidase in oxygen-dependent killing of Staphylococcus aureus by neutrophils. Infect Immun..

[13] Van den Berg J.M., van Koppen E., Ahlin A., Belohradsky B.H., Bernatowska E., Corbeel L., Espanol T., Fischer A., Kurenko-Deptuch M., Mouy R. (2009). Chronic granulomatous disease: The European experience. PLoS ONE.

[14] De Souza Ferreira C., Araújo T.H., Ângelo M.L., Pennacchi P.C., Okada S.S., de Araujo Paula F.B., Migliorini S., Rodrigues M.R. (2012). Neutrophil dysfunction induced by hyperglycemia: Modulation of myeloperoxidase activity. Cell. Biochem. Funct..

[15] Ahmed M.U., Brinkmann Frye E., Degenhardt T.P., Thorpe S.R., Baynes J.W. (1997). N-epsilon-(carboxyethyl)lysine, a product of the chemical modification of proteins by methylglyoxal, increases with age in human lens proteins. Biochem. J..

[16] Marchetti C., Patriarca P., Pietro Solero G., Baralle F.E., Romano M. (2004). Genetic Characterization of Myeloperoxidase Deficiency in Italy. Hum. Mut..

[17] MacCarthy K.G., Dahl M.V. (1989). Inhibition of growth of Trichophyto rubrum by the myeloperoxidase-hydrogen peroxide-chloride system. J. Investig. Dermatol..

[18] Nguyen C., Katner H.P. (1997). Myeloperoxidase deficiency manifesting as pustular candida dermatitis. Clin. Infect. Dis..

[19] Parry M.F., Root R.K., Metcalf J.A., Delaney K.K., Kaplow L.S., Richar W.J. (1981). Myeloperoxidase deficiency: Prevalence and clinical significance. Ann. Intern. Med..

[20] Weber M.L., Abela A., de Repentigny L., Garel L., Lapointe N. (1987). Myeloperoxidase deficiency with extensive candidal osteomyelitis of the base of the skull. Pediatrics.

[21] El-Benna J., Dang P.M.C., Gougerot-Pocidalo M.A., Marie J.C., Braut-Boucher F. (2009). p47phox, the phagocyte NADPH oxidase/NOX2 organizer: Structure, phosphorylation and implication in diseases. Exp. Mol. Med..

[22] De Leo F.R., Ulman K.V., Davis A.R., Jutila K.L., Quinn M.T. (1996). Assembly of the human neutrophil NADPH oxidase involves binding of p67phox and flavocytochrome b to a common functional domain in p47phox. J. Biol. Chem..

[23] Dang P.M., Cross A.R., Babior B.M. (2001). Assembly of the neutrophil respiratory burst oxidase: A direct interaction between p67PHOX and cytochrome b558. Proc. Natl. Acad. Sci. USA.

[24] Nisimoto Y., Motalebi S., Han C.H., Lambeth J.D. (1999). The p67(phox) activation domain regulates electron flow from NADPH to flavin in flavocytochrome b (558). J. Biol. Chem..

[25] Jackson S.H., Gallin J.I., Holland S.M. (1995). The p47phox mouse knock-out model of chronic granulomatous disease. J. Exp. Med..

[26] Denson L.A., Jurickova I., Karns R., Shaw K.A., Cutler D.J., Okou D.T., Dodd A., Quinn K., Mondal K., Aronow B.J. (2018). Clinical and Genomic Correlates of Neutrophil Reactive Oxygen Species Production in Pediatric Patients With Crohn’s Disease. Gastroenterol.

[27] Bielinski S.J., Hall J.L., Pankow J.S., Boerwinkle E., Matijevic-Aleksic N., He M., Chambless L., Folsom A.R. (2011). Genetic variants in TLR2 and TLR4 are associated with markers of monocyte activation: The Atherosclerosis Risk in Communities MRI Study. Hum. Genet..

[28] Lek L., Karczewski K.J., Minikel E.V., Samocha K.E., Banks E., Fennell T., O’Donnell-Luria A.H., Ware J.S., Hill A.J., Cummings B.B. (2016). Analysis of protein-coding genetic variation in 60,706 humans. Nature.

[29] D’Ostiani C.F., Del Sero G., Bacci A., Montagnoli C., Spreca A., Mencacci A., Ricciardi-Castagnolic P., Romania L. (2000). Dendritic cells discriminate between yeast and hyphae of the fungus Candida albicans: Implications for initiation of T Helper immunity in vitro and in vivo. J. Exp. Med. N. Y..

[30] Blake J.S., Dahl M.V., Herron M.J., Nelson R.D. (1991). An immunoinhibitory cell wall glycoprotein (mannan) from Trichophyton rubrum. J. Investig. Dermatol..

